# Sex, Allergic Diseases and Omalizumab

**DOI:** 10.3390/biomedicines10020328

**Published:** 2022-01-29

**Authors:** Maria Maddalena Sirufo, Francesca De Pietro, Lia Ginaldi, Massimo De Martinis

**Affiliations:** 1 Department of Life, Health and Environmental Sciences, University of L’Aquila, Piazzale Salvatore Tommasi n.1, 67100 L’Aquila, Italy; maddalena.sirufo@gmail.com (M.M.S.); fra722@hotmail.it (F.D.P.); demartinis@cc.univaq.it (M.D.M.); 2 Allergology and Clinical Immunology Unit, ASL 04, 64100 Teramo, Italy

**Keywords:** allergy, omalizumab, urticaria, asthma, IgE, gender medicine, biological therapy

## Abstract

Gender differences are increasingly emerging in every area of medicine including drug therapy; however, specific gender-targeted studies are infrequent. Sex is a fundamental variable, which cannot be neglected. When optimizing therapies, gender pharmacology must always be considered in order to improve the effectiveness and safety of the use of drugs. Knowledge of gender differences promotes appropriate use of therapies and greater health protection for both genders. Further development of gender research would make it possible to report on differences in the assimilation and response of the female organism as compared to the male, in order to identify potential risks and benefits that can be found between genders. Furthermore, a better understanding of sex/gender-related influences, with regard to pharmacological activity, would allow the development of personalized “tailor-made” medicines. Here, we summarize the state of knowledge on the role of sex in several allergic diseases and their treatment with omalizumab, the first biologic drug authorized for use in the field of allergology.

## 1. Introduction

The differences between the sexes affect both physiological and pathological aspects of human health [1]. The immune system and inflammation both differ between men and women, which impacts the progression, severity, incidence and therapy of diseases [2,3]. Certain side effects of drugs are reported more in women than in men; sex-related differences have been analyzed in terms of pharmacokinetics and pharmacodynamics, revealing differences in therapeutic efficacy and side effects [4]. Unfortunately, while there are well-described differences between the sexes, the underlying mechanisms are far from clear. In general, women exhibit higher immunoglobulin levels and a better immune response after immunization or infection, alongside a greater tendency to develop chronic pain and autoimmune pathologies [5]. These sex disparities seem to be caused by the X and Y chromosomes and sex hormones (testosterone, progesterone and estradiol) [6,7,8,9,10,11,12].

## 2. Sex Differences in Autoimmune Diseases, Inflammatory Disorders and Allergy

The prevalence and onset of pathologies changes according to sex hormones [13,14,15]. Sex steroids, in fact, affect the distribution and function of both innate and adaptive immune cells, leading to immune and inflammatory responses which differ between the sexes [16,17,18,19]. The female sex hormones estrogen and progesterone, as well as the male androgens, elicit direct effects on the function of immune cells. The existence of sex-specific transcriptomes and methylomes suggests that there is a difference between the sexes even at the epigenetic level [20].

Estrogen has been implicated in neutrophil apoptosis and the formation of neutrophil extracellular traps capable of binding pathogens, resulting in cell death [21]. In contrast to estrogen, progesterone has been shown to have a suppressive effect on the formation of neutrophil extracellular traps [22]. A reduction in spontaneous apoptosis was observed in female-derived neutrophils, as compared to those derived from males [23]. The X chromosome is thought to be involved in the better immune responses of women, making them more resistant to infections, and in the breakdown of self-tolerance which is observed as a predominance of autoimmune diseases in women (78%) [24]. Estrogens possess inductive effects on autoimmune-related immune responses and are considered to promote inflammatory reactions. Female sex hormones, estrogens, promote the autoimmune response and are considered pro-inflammatory. However, estrogens may exhibit anti-inflammatory features depending on the cell types involved, the immune stimulus, target organ, concentration, timing and intracellular metabolism of the estrogen, and estrogen receptor subtype expression. On the other hand, male sex hormones perform immunosuppressive and anti-inflammatory activities. Scleroderma, multiple sclerosis, rheumatoid arthritis and asthma, in particular, reflect these gender differences [25,26,27,28,29,30]. The incidence and course of systemic lupus erythematosus (SLE) differ between the sexes, with a nine-fold higher incidence in women linked to the influence of estrogen on epigenetic, immune and genetic factors (X-linked genes such as Foxp3, TNF and Tlr7) [31,32]. Hashimoto’s thyroiditis, Graves’ disease, myasthenia gravis, multiple sclerosis and Sjögren’s syndrome are also prevalent in women compared to men [33,34,35,36,37]. Gender differences have been documented in patients with Crohn’s disease, with more girls affected by extra-intestinal manifestations and subject to ileocecal resections than boys [38,39,40]. In contrast, the reverse relationship occurs for cardiovascular disease (CVD), with men more at risk than women of the same age, reinforcing the protective role of estrogen in these pathologies [41,42]. Women also show better resolution of sepsis episodes and have a lower incidence of gout in the fertile period than men [43,44]. The perception of pain is more intense in women [9]; this could make it difficult to identify the severity of the pathology, as happens in female asthmatic patients who describe more symptoms and a worse quality of life [45,46,47,48,49,50]. Muñoz-Cruz S. et al. demonstrated that estradiol, progesterone, testosterone and DHT regulate the release of histamine by mast cells (MC) via binding to receptors present on MCs in dose- and gender-dependent manners [51].

In addition, the activity of hormones in atopic dermatitis (AD) has been demonstrated [52], with a different dermatological manifestation based on IgE sensitization only in male patients [53].

Male sex hormones, testosterone and dehydroepiandrosterone appear to be reduced in dermatological patients, while estrogens are able to activate mast cells and influence the female prevalence of urticaria, especially in certain age groups [54]. Conversely, hormones do not appear to have a significant effect on the outcome of the oral food challenge (OFC) [55].

## 3. Asthma and Hormone-Related Differences

Asthma is a common, heterogenous, multifactorial and chronic inflammatory airway disease, characterized by reversible bronchial obstruction, bronchial hyper-responsiveness and airway inflammation. Asthma affects more than 300 million people, with a prevalence of 6% in adults and 4% in minors [56]. Asthmatic adult females have a predominant prevalence: 9.7% versus 5.7% in males, a predominance of non-allergic asthma and a more severe morbidity than males, such as more visits and hospitalizations and higher death rates [57]. Approximately 5–10% of the population is affected by asthma, often adequately controlled by corticosteroid therapy (ICS) and long-acting beta2-agonists (LABA) despite increased anxiety and depression in these patients [58,59]. Elderly patients (>65 years) represent a class that is most at risk due to the presence of comorbidities, therapies already in place and technical difficulties in handling inhalers [60]. These patients are also likely to be underdiagnosed, due to factors such as a tendency to minimize symptoms, technical difficulties in performing spirometry diagnoses and a lack of adapted reference values for functional test results [61]. The different prevalence of asthma in the two sexes and the increased incidence in women in the reproductive years would seem to suggest a protective role of androgens compared to the pro-inflammatory one played by estrogens [62]. In particular, an increased production of leukotrienes was observed in women which is potentially responsible for the increase in the incidence and severity of the disease in women [9]. The reported trends in asthma prevalence suggest that sex hormones are implicated in asthma pathogenesis, with female sex hormones and their receptors favoring asthma development, and male sex hormones and their receptors exerting a protective effect. Some authors have suggested a greater hyper-responsiveness in females than in males, others have underlined differences between genders in lung capacity [57]. Gender differences are found both in the anatomy and in the perception of the pathology. Female lungs are smaller and have fewer alveoli but show better forced expiratory flows. Despite this, the perception of the disease is worse in women with a greater impact on quality of life (QoL) [58]. The prevalence and severity of asthma are frequently associated with key moments in the reproductive life of women, with worse symptoms during the cycle and the premenstrual phases [63]. The hormonal fluctuations characterizing these phases determine a greater bronchial hyper-reactivity through an increase in the inflammatory response, with a higher incidence of severe asthma during menstruation. In particular, in women with severe asthma, some markers of inflammation including eosinophils in the sputum, exhaled nitric oxide and serum concentrations of leukotrienes and C4 are increased [64,65]. Senna et al. showed that respiratory allergies, especially asthma, but also allergic rhinitis, are prevalent in males in childhood but occur at a higher rate in the female sex in adulthood [66]. This difference between females and males is based on the immunological effects of female hormones and particularly their capacity of modulation of the inflammatory response. Estrogens act on several cells: dendritic cells, innate lymphoid cells, Th1, Th2, T_reg_ and B_regs_ cells, and on a number of cytokines, including interleukin (IL)-4, IL-5, IL-10 and IL-13, producing increased IgE-dependent and IgE-independent degranulation, increased DC differentiation, increased cytokine responsiveness, production of IL-8, IL-6, IL-4, Il-5, IL-13, IL-10 and MCP-1, up-regulation of CD40 and MHCII, enhancement of the type 2 response, suppression of the type 1 response, increased expression of histamine receptors 2 and 3, down-regulation of IL-33 and a reduced susceptibility to allergic airway inflammation [54,67,68,69,70,71,72]. A higher risk of new-onset asthma or exacerbations has been reported in women using postmenopausal hormone replacement therapy. Matteis et al. showed that in 30% of asthmatic women, there was a marked bronchial reactivity to methacholine during the follicular phase. Conversely, in the luteal phase, there were higher serum and sputum testosterone levels with lower bronchial reactivity compared to the follicular phase. No impact of sex on response to therapy in asthmatic individuals was reported by the Asthma Clinical Research Network (ACRN) of the National Heart, Lung and Blood Institute [73]. Treatment failures and the use of rescue drugs are more frequent in women, although this observation is not statistically significant. FEV1 percentages were found to be slightly higher in females than in males, while no significant differences were reported for IgE levels, blood eosinophils, exhaled nitric oxide levels or symptoms. It was also shown that there was no difference between women and men over the age of 30 with regard to treatment failure. Instead, gender differences were found in personality traits and beliefs regarding therapy, which could influence therapeutic adherence in asthmatic patients [74].

Females had better respiratory function but reported more nocturnal awakenings and difficulties in daily activities [75]. Therapeutic outcomes and perception of the disease vary according to gender. Further studies to understand the mechanisms underlying these differences would be useful to ensure a personalized diagnostic approach and gender-oriented therapy [58].

## 4. Urticaria

Urticaria is an itchy skin disease characterized by raised, circumscribed, erythematous, edematous and unprompted wheals. Wheals with varying distribution are generally circular and evanescent over a few hours. Urticaria is classified as acute if it lasts less than 6 weeks or chronic (CU) if the manifestation exceeds 2 weeks. In turn, CU is divided into chronic spontaneous urticaria (CSU) and physical urticaria (CINDU): lesions in patients with CSU occur spontaneously, without physical or environmental stimuli, whereas CINDU is less common and requires specific triggers which can be physical stimuli. Eun S.J. et al. [54] reported that male sex, being over 10 years of age, urban residency and autoimmune thyroid disease were associated with an increased risk of chronicity. Urticaria is more common in women, and autoimmune conditions often underlie the onset of CSU, with autoimmune thyroid disease associated with a higher incidence and chronicity of urticaria [76]. In fact, CU can also be a manifestation associated with autoimmune diseases, such as systemic lupus erythematosus, rheumatoid arthritis, hypo/hyperthyroidism, Sjögren’s syndrome, type 1 diabetes mellitus, celiac disease, some auto-inflammatory syndromes and malignancy. However, recent studies have revealed that a female predominance of urticaria is observed only in specific age groups. A clear female predominance for new-onset urticaria is reported only for patients aged 20–44 and 45–64 years [77]. The female predominance of urticaria in specific age groups could be due to estrogen. Estrogen is believed to enhance humoral immunity and antibody synthesis. The fact that CU is twice as frequent in women than in men may be associated with diseases and conditions characterized by sex hormone changes, including those caused by hormonal contraceptives, pregnancy, the menstrual cycle, menopause and/or hormone replacement therapy. In light of these facts, Rogala et al. suggested that fluctuations in the hormonal milieu may play a role in the pathogenesis of the disease. In support of this hypothesis is the fact that, as with estradiol, low concentrations of environmental estrogens are capable of causing mast cell degranulation, suggesting they play a role in the pathogenesis of mast cell-dependent diseases [78]. A gender difference in the expression profiles of histamine receptors and mast cells was also demonstrated in experimental studies [79,80].

## 5. Sex and Biological Therapy

The effectiveness of some drugs differs between the two sexes, and yet, current treatments are not personalized to the patient, affecting both women and men without the evaluation of the effectiveness of the drug by gender.

Gender differences in pharmacology are very important and partly attributable to the different biology of the two sexes. Hormonal variations, weight, body composition, gastric acidity and glomerular filtration can influence the absorption, distribution, metabolism and elimination of drugs.

Although sex is reported in all studies, subgroup analyses are only rarely undertaken, except for when pathologies show a clear sex-dependent phenotype [81]. Similarly, whether, and which, adverse drug effects are prevalent in men or women should be assessed in the registration of studies, considering the different metabolic responses to drugs in absorbing and eliminating the active molecules contained within them.

In recent years, significant differences have been documented between women and men in response to biologic drugs used in the therapy of neoplastic and chronic inflammatory diseases, such as rheumatic diseases and chronic inflammatory bowel diseases (Table 1). An appropriate and effective treatment of autoimmune diseases requires personalization that must include gender analysis, as also suggested for systemic sclerosis [82,83].

Cutolo et al. [84] evaluated patients with rheumatoid arthritis treated with anti-TNF therapies and observed the effect of these drugs on the balance of sex hormone production. The results of the ANTARES study of anti-TNF therapies suggest that TNF-blocking drugs work by blocking the conversion of hormones from androgens (anti-inflammatory) to estrogens (pro-inflammatory) in the synovium of RA patients. In fact, testosterone, according to in vitro studies, is able to increase the expression of the anti-inflammatory cytokine IL-10 and reduce the production of pro-inflammatory cytokines [84,85]. Among men, better responses to anti-TNF therapy are reported compared to women [86]. A 12-year persistence and male gender are both predictors of failure of the initial biologic therapy in patients with rheumatoid arthritis and psoriatic arthritis [87], while a lower efficacy of anti-TNF drugs is observed in females with axial spondyloarthritis [88]. Furthermore, there is a recognized sex bias in the treatment of rheumatic diseases with biologics [89,90]. Initiation of anti-TNF-α therapy in Crohn’s disease results in a rapid and significant increase in gonadotropin levels and sex hormones, together with a reduction in inflammation [91]. In oncology, it has been demonstrated that reducing testosterone levels can improve the efficacy of immunotherapy [92]. In allergology, there are several biologic drugs currently authorized, but very little is known about potential gender differences in their efficacy and safety [93,94].

## 6. Sex Difference in Omalizumab Therapy

Biologics have recently been approved for the treatment of asthma which has been poorly controlled by first-line therapy.

These drugs, although expensive, are preferable to the prolonged use of systemic steroids in order to avoid side effects [95]. Milger et al. reported that women were more frequently treated with biological therapies than men. The increase in steroid use with age goes in parallel with increasing disease severity as measured by symptoms and lung function. The frequent use of systemic steroids should be avoided in preference of antibody therapies, in order to reduce the side effects [94].

**Figure 1:**
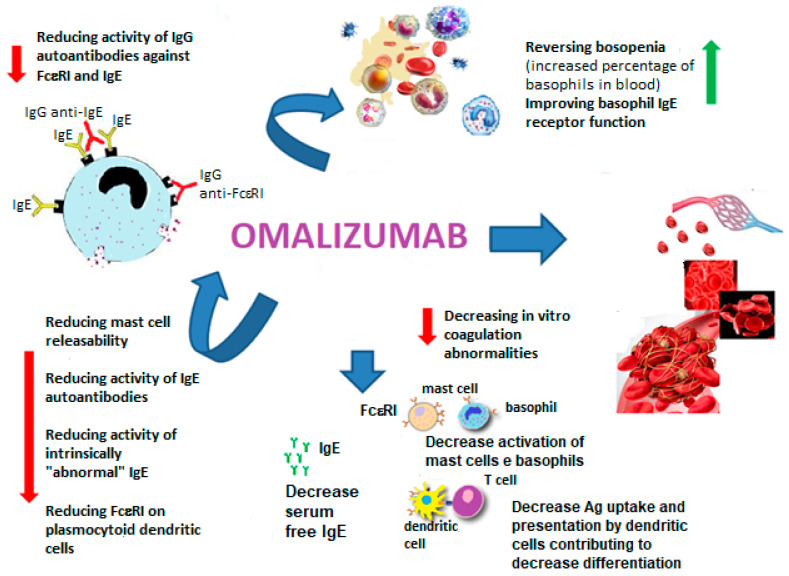
Omalizumab works by: reducing the activity of IgG autoantibodies against FcεRI and IgE, reducing mast cell releasability, improving basophil IgE receptor function, reversing basopenia (increased percentage of basophils in the blood), reducing the activity of IgE autoantibodies, reducing the activity of intrinsically “abnormal” IgE, decreasing in vitro coagulation abnormalities and reducing the amount of FcεRI on plasmacytoid dendritic cells.

Habitual smoking was less present in asthmatic women than in men, resulting in better lung function [56]. One biological therapy is omalizumab (OmAb), a recombinant DNA-derived humanized immunoglobulin G1K monoclonal antibody that selectively binds to free human IgE and pre-bound IgE [96] (Figure 1).

Omalizumab (OmAb) is approved for the treatment of treatment-resistant allergic asthma and CIU/CSU. In particular, as allergic asthma is most prevalent in childhood, most children with severe asthma are eligible for treatment with OmAb, for which there is a greater wealth of experience in children than for anti-eosinophilic antibodies [96,97,98].

Although not yet authorized for wider use, omalizumab also appears effective in the treatment of other diseases such as atopic dermatitis and food allergies [99,100,101,102,103,104] (Table 2).

Treatment with OmAb improves the management of the disease and related symptoms, reducing rates of depression by 60% compared to inadequately controlled asthmatics [105].

Nevertheless, Canonica et al. [106] reported a gender difference in disease perception that appeared significantly worse in females after one year of therapy. In particular, some aspects of the disease such as symptom experience, level of concern about asthma and emotional impact were perceived to be significantly worse in females. The role of sex disparities is well recognized in asthma. International guidelines encourage the analysis of clinical data according to sex and gender, or both where appropriate, as gender bias is detectable in the design of published clinical trials. Omalizumab is the first biologic drug launched for asthma, and its trials began with a smaller proportion of women. Only in the two most recent trials of omalizumab was an analysis by sex performed [107,108,109,110,111]. It is therefore important to consider sex as a variable when evaluating the response, as well as the other indicators, in order to understand if this can provide additional predictive information.

Omalizumab is also a safe and effective drug in the treatment of CSU in patients who are resistant to second-generation H1 antihistamine therapy [112]. The drug induces eosinophil apoptosis and inhibits T cell activation with an anti-inflammatory effect [115,116]. Recent reports of the treatment of CSU with omalizumab describe a better response in women than in men. With respect to sex and recurrence, there is no correlation with age, body mass index, peripheral eosinophil counts, total IgE levels, D-dimer levels, plasma prothrombine levels or C-reactive protein, and no sex differences in tolerability or safety are observed [113]. In contrast, other authors found no significant differences in sex, age and thyroid autoimmunity between responders and non-responders in CSU patients treated with omalizumab [114]. The pathogenic role played by female hormones in CU and allergic asthma, both of which exhibit a good response to omalizumab and high prevalence in females, highlights the possible action of omalizumab directly on sex hormones, or indirectly on the downstream mechanisms they mediate. Further studies would be useful in order to better understand whether omalizumab acts differently between women and men and whether hormones are implicated in the mechanism of action.
**A summary of the current knowledge of the role of sex in the action and efficacy of omalizumab is presented below:**Omalizumab is the first biologic therapy launched for asthma, its trials began with a smaller proportion of women and only the two most recent trials of omalizumab included an analysis by sex.Although asthma symptoms improve following omalizumab treatment, the overall perception of asthma is significantly worse in females compared to males at a 12-month follow-up visit.In the treatment of CSU, omalizumab may obtain a better response in women than in men.There are no sex differences in the tolerability and safety of omalizumab.A possible action of omalizumab on sex hormones, or indirectly on the downstream mechanisms mediated by them, can be hypothesized.

## 7. Conclusions

Although the National Institutes of Health (NIH) of the United States indicated in 2014 that gender should be taken into account in studies and considered a biological variable, to date, there is still a long way to go on the topic. Gender medicine does not represent a separate branch of the medical field but an interdisciplinary dimension which, as such, must pervade all branches of medical knowledge in order to study the influence of sex and gender on human physiology and pathology. That is to say, sex and gender influence how pathologies develop and what the symptoms are, as well as prevention, diagnosis and therapy, in men and women. Among the various subspecialties of medicine, allergology and the most modern biotechnological treatments are particularly lacking from the point of view of gender studies. There is evidence of the importance of gender differences in allergic diseases and their treatment; however, research in this area is still in its infancy and there are fundamental gaps that need to be filled. It is not yet clear whether or not sex influences the action of omalizumab in its therapeutic uses; the literature reports conflicting data, although the key role of sex hormones in the prevalence and in some pathogenetic aspects of the mentioned pathologies necessitates further study in this field. We believe it is essential to investigate how allergic diseases differ between men and women in terms of prevention, clinical signs, therapeutic approach, prognosis and psychological and social impact. It is also necessary to pay particular attention to reproductive aspects, such as pregnancy and breastfeeding. For this purpose, it would be useful to: create a network of epidemiological datasets, specific for each condition and treatment, and platforms for sharing data; reassess the validity of clinical tests and scales currently in use, in the light of gender differences; conduct a thorough analysis of sex differences in biomarker data in large datasets, as well as the interaction of sex with clinical progression and other biomarkers; and ensure adequate representation of males and females in studies.

## Figures and Tables

**Table 1:** Bibliography on sex differences in response to biological therapy.

Reference	Title	Journal
[34]	Sex and Management of Rheumatoid Arthritis.	*Clin. Rev. Allergy Immunol.* 2019
[84]	Anti-TNF and sex hormones.	*Ann. N. Y. Acad. Sci.* 2006
[85]	Gender Differences in Rheumatoid Arthritis: Interleukin-4 Plays an Important Role.	*J Immunol Res.* 2020
[86]	Sex differences in response to anti-tumor necrosis factor therapy in early and established rheumatoid arthritis—results from the DANBIO registry.	*J. Rheumatol.* 2012
[87]	Long-term remission and biologic persistence rates: 12-year real-world data.	*Arthritis Res. Ther.* 2021
[88]	Body weight, gender and response to TNF-α blockers in axial spondyloarthritis.	*Rheumatology* (Oxford). 2014
[89]	Is there a sex bias in prescribing anti-tumour necrosis factor medications to patients with rheumatoid arthritis? A nation-wide cross-sectional study.	*Ann. Rheum. Dis.* 2012
[90]	Sex Differences in the Treatment of Psoriatic Arthritis: A Systematic Literature Review.	*Isr. Med. Assoc. J.* 2016
[91]	Increases in Sex Hormones during Anti-Tumor Necrosis Factor α Therapy in Adolescents with Crohn’s Disease.	*J. Pediatr.* 2016
[92]	Downregulating testosterone levels enhance immunotherapy efficiency.	*Oncoimmunology* 2021
[93]	Strategies for choosing a biologic for your patient with allergy or asthma.	*Ann. Allergy Asthma Immunol.* 2021
[94]	Targeted Molecular Therapies in Allergy and Rhinology.	*Otolaryngol. Head Neck Surg.* 2021

**Table 2:** Bibliography on sex differences in allergy and response to omalizumab.

Reference	Title	Journal
[95]	Asthma over the Adult Life Course: Gender and Hormonal Influences.	*Clin. Chest Med.* 2019
[96]	Age- and sex-dependent differences in patients with severe asthma included in the German Asthma Net cohort.	*Respir. Med.* 2020
[97]	Translational Allergy and Omalizumab: The Pioneer.	*Journal of Pharmaceutical Education and Research* 2021
[98]	Successful Treatment With Omalizumab in a Child With Asthma and Urticaria: A Clinical Case Report.	*Front. Pediatr.* 2019
[99]	Omalizumab an effective and safe alternative therapy in severe refractory atopic dermatitis: A case report.	*Medicine* (Baltimore). 2018
[100]	Food Allergy Insights: A Changing Landscape.	*Arch. Immunol Ther. Exp.* (Warsz.) 2020
[101]	New Perspectives in Food Allergy.	*Int. J. Mol. Sci.* 2020
[102]	Food Allergies and Ageing.	*Int. J. Mol. Sci.* 2019
[103]	Omalizumab effectiveness in patients with a previously failed oral immunotherapy for severe milk allergy.	*Immun. Inflamm. Dis.* 2021
[104]	Appraisal of the Real-World Effectiveness of Biologic Therapies in Aspirin-Exacerbated Respiratory Disease.	*J. Allergy Clin. Immunol. Pract.* 2021
[105]	Benefits of omalizumab on anxiety and depression in patients with severe asthma.	*Caspian J. Intern. Med.* 2018
[106]	Improvement of patient-reported outcomes in severe allergic asthma by omalizumab treatment: the real life observational PROXIMA study.	*World Allergy Organ J.* 2018
[107]	Omalizumab Improves Quality of Life and Asthma Control in Chinese Patients With Moderate to Severe Asthma: A Randomized Phase III Study.	*Allergy Asthma Immunol. Res.* 2016
[108]	Factors reducing omalizumab response in severe asthma.	*Eur. J. Intern. Med.* 2018
[109]	Obesity influences the outcomes of anti-IgE (omalizumab) therapy of asthma.	*Clin. Exp. Allergy* 2020
[110]	Gender bias in clinical trials of biological agents for severe asthma: A systematic review.	*PLOS One* 2021
[111]	Sex differences in the efficacy, safety, and tolerability of omalizumab after 1 year in Maltese patients with asthma.	*Ann. Allergy Asthma Immunol.* 2017
[112]	Solar Urticaria, a Disease with Many Dark Sides: Is Omalizumab the Right Therapeutic Response? Reflections from a Clinical Case Report.	*Open Med.* (Wars.) 2019
[113]	Sex differences in the efficacy of Omalizumab in the treatment of chronic spontaneous urticaria.	*Int. J. Immunopathol. Pharmacol.* 2021
[114]	Analysis of clinical factors as possible predictors of response to omalizumab and relapse after treatment discontinuation in chronic spontaneous urticaria.	*Dermatol. Ther.* 2021
